# Valorization of Olive Pomace into Functional Hydrochars for Dye Removal from Water: Effects of Hydrothermal Carbonization and Soft Alkaline Activation

**DOI:** 10.3390/molecules31152617

**Published:** 2026-07-27

**Authors:** Gianluigi Farru, Gennaro Pace, Antonio Di Virgilio, Fabiano Asunis, Angela De Bonis, Maria Cristina Mascolo, Salvatore Masi, Francesco Di Capua

**Affiliations:** 1 Department of Civil and Environmental Engineering and Architecture, University of Cagliari, Via Marengo 2, 09123 Cagliari, Italy; gianluigi.farru@unica.it (G.F.); fabiano.asunis@unica.it (F.A.); 2 Department of Engineering, University of Basilicata, Via dell’Ateneo Lucano 10, 85100 Potenza, Italy; gennaro.pace@unibas.it (G.P.); salvatore.masi@unibas.it (S.M.); 3 Department of Basic and Applied Science, University of Basilicata, Via dell’Ateneo Lucano 10, 85100 Potenza, Italy; angela.debonis@unibas.it; 4 Department of Letter and Philosophy, University of Cassino and Southern Lazio, Via S. Angelo—Località Folcara, 03043 Cassino, Italy; mc.mascolo@unicas.it; 5 Materials Laboratory, Department of Civil and Mechanical Engineering, University of Cassino and Southern Lazio, Via Gaetano di Biasio 43, 03043 Cassino, Italy; 6 Institute of Environmental Geology and Geoengineering, National Research Council, Area della Ricerca di Roma 1, Via Salaria Km 29.300, C.P. 10, Monterotondo Stazione, 00015 Rome, Italy

**Keywords:** methylene blue, agro-industrial residues, carbonaceous sorbents, surface functionalization, pore accessibility

## Abstract

Olive pomace (OP), an abundant byproduct of the olive oil industry, was investigated as a low-cost precursor for the production of hydrochar-based adsorbents for methylene blue (MB) removal from water. Hydrothermal carbonization (HTC) was performed at 180 and 220 °C with different residence times, followed by soft alkaline activation (SAA), consisting of alkaline treatment and carbonization at 300 °C, and post-washing to improve the adsorption performance. The materials were characterized by ATR-FTIR, Raman spectroscopy, XRD, SEM-EDX, BET, and mercury intrusion porosimetry. HTC increased the degree of carbonization and surface area, but non-activated hydrochars showed lower adsorption capacity than untreated OP because of the loss of oxygenated functional groups. Among non-activated hydrochars, OP_220_1 showed the highest adsorption capacity (20.7 mg g^−1^). SAA increased the adsorption capacity of the hydrochar by 63%, while washing further enhanced performance by removing carbonate precipitates and restoring pore accessibility. The washed activated hydrochar derived from OP_220_1 achieved the highest adsorption capacity (69.9 mg g^−1^ at 250 mg L^−1^ MB) and BET surface area (40.0 m^2^ g^−1^). Kinetics followed the pseudo-second-order model, while washing shifted the isotherm behavior from Langmuir to Freundlich. A performance-based assessment showed that the washed activated hydrochar reduced adsorbent demand by 49% compared with untreated OP.

## 1. Introduction

Olive pomace (OP), a byproduct of olive oil production, has emerged as an important material due to its diverse composition and potential for sustainable applications. Olive oil production is a key industry in the Mediterranean region and generates a significant amount of waste, with OP accounting for approximately 35–40% of the total weight of the olives processed. This byproduct is made up of olive skin, pulp, seeds, and residual water, and its composition varies depending on the extraction method and the type of olives used [1]. OP is rich in organic compounds such as fibers, phenolic compounds, and minerals, with a high content of carbon, making it a valuable material for various applications, including energy production, recovery of bioactive compounds, and the formulation of organic fertilizers [2].

The total global production of waste biomass generated from the extraction of olive oil is estimated to be at least 40 Mt year^−1^, with OP accounting for more than 20 Mt year^−1^ [3]. The European Union (EU) is the world’s largest olive oil producer, accounting for around 2/3 of the world production [4], resulting in the generation of up to 10 Mt year^−1^ of OP, which corresponds to nearly half of the global OP production. This results in a large volume of waste that poses significant challenges in terms of disposal. Consequently, interest in the valorization of OP for sustainable applications has increased over the years.

The utilization of OP has traditionally been limited to animal feed, composting, and energy production through direct combustion or biogas generation [1]. However, among the different valorization routes, recent research has increasingly focused on expanding its applications, particularly in the environmental sector [5]. Dye-containing wastewater can be treated by biological processes, coagulation–flocculation, membrane separation, advanced oxidation, and adsorption, although these methods may be limited by poor dye biodegradability, sludge production, membrane fouling, or high chemical and energy demand. Adsorption is therefore attractive because of its operational simplicity, high efficiency, and the possibility of using low-cost waste-derived adsorbents. OP and its carbonized derivatives have been investigated for the removal of environmental contaminants, particularly through adsorption [6,7,8]. Research has demonstrated that OP itself can serve as an effective adsorbent for pollutants due to its high organic content and natural adsorption sites. For example, Abdoul-Latif et al. [7] found that unprocessed OP could adsorb up to 21.3 mg g^−1^ of total phenols from olive mill effluent, while OP biochar showed an even higher adsorption capacity of 66.7 mg g^−1^. Similarly, OP has shown promise in adsorbing heavy metals, such as iron and manganese, from aqueous solutions with adsorption capacities of about 10 mg g^−1^ [9].

Carbonized derivatives of OP, such as biochar and activated carbon, can exhibit significantly enhanced adsorption capacities. The pyrolysis process used to produce biochar can increase its surface area to 3–6 m^2^ g^−1^ and create mesopores, thereby improving its adsorption efficiency [10]. Activated carbon produced from OP through physicochemical treatment exhibits a much higher specific surface area than biochar (>1600 m^2^ g^−1^), which can enhance its adsorption capacity, but its production has been reported to yield high amounts of volatile organic and sulfur compounds, which is a disadvantage from an environmental perspective. Moreover, the production of activated carbon includes mechanical pre-activation, thermochemical treatment, and chemical purification, which results in a relatively high production cost (~10 € kg^−1^) despite the feedstock being waste material [11]. Therefore, a more cost-effective production process is needed to promote the use of OP and its derivatives as adsorbent materials.

Recently, soft alkaline activation (SAA), consisting of alkaline pretreatment followed by low-temperature (300 °C) carbonization, has been proposed as a cost-effective and environmentally sustainable activation process for organic waste materials such as spent coffee grounds [12]. Olive-processing residues [6,7,8,9,10,11] and spent coffee grounds [12] represent relevant examples of agro-industrial wastes investigated as low-cost adsorbent precursors. SAA led to the generation of a promising low-cost adsorbent material, with adsorption performances comparable to those of activated carbon materials. To date, SAA has never been applied to OP and its carbonized derivatives. Therefore, the research question regarding the impact of this activation process on the adsorption capacity of these materials remains open.

In addition to pyrolysis, hydrothermal carbonization (HTC), another thermochemical conversion process, has gained increasing attention in recent years due to its potential benefits such as lower temperatures (180–250 °C) and the ability to directly process wet organic materials without the energy-intensive drying stage required in pyrolysis. This lowers the operational costs of the process significantly (>10-fold cost reduction has been estimated) and may reduce the selling price of the resulting carbonaceous solid, i.e., hydrochar, thereby promoting its commercialization and supporting circular-economy applications [13]. Following the growing interest in HTC and its products, hydrochars have been investigated as adsorbent materials for the removal of various contaminants, including heavy metals, organic dyes, herbicides/pesticides, and pharmaceuticals [14]. In the literature, only a few studies exist on the utilization of hydrochar from olive residues as adsorbent materials. González & Manyà [15] investigated CO_2_ adsorption using physically and thermally activated hydrochars from olive mill waste, while Chatir et al. [16] produced the hydrochar from powdered olive stones mixed with water and treated at 200 °C for 6 h and tested it for the removal of diclofenac. Capobianco et al. [17] developed a low-cost iron-coated adsorbent from olive pomace processed via HTC for arsenic removal and showed that alkaline conditions yield the highest arsenic uptake. Haris et al. [18] utilized one type of hydrochar derived from an Australian three-phase olive mill waste for the removal of azo and non-azo dyes from water, showing promising adsorption capacity for single and binary adsorption. The physicochemical properties of hydrochar materials depend on many factors, including the feedstock material, temperature, residence time, pressure, reacting atmosphere, catalysts, biomass loading and pH of reaction. Therefore, fine-tuning HTC operation can lead to the production of a more suitable hydrochar for adsorption purposes.

Although OP and OP-derived biochars have been investigated for adsorption applications, the use of HTC-derived OP hydrochars remains comparatively less explored, and the effect of HTC operating conditions on adsorption performance has not been systematically clarified. Moreover, to the best of our knowledge, SAA has not yet been applied to OP-derived hydrochars, and the role of post-washing has not been investigated.

This study investigates the adsorption performance of raw OP and derived hydrochars produced under different HTC operating conditions (temperature and residence time) using methylene blue (MB) as a model pollutant commonly found in textile industry wastewater. Unlike previous studies, the present work systematically evaluates the role of HTC severity, SAA and washing on surface chemistry, porosity and adsorption efficiency. The influence of HTC operational settings on adsorption capacity and kinetics was investigated in batch adsorption tests and through physicochemical characterization of the produced hydrochars. The best adsorbent materials were further characterized in terms of isotherm and thermodynamic behaviour. Performance-based techno-economic considerations were also included to evaluate the potential large-scale use of OP-derived hydrochars as adsorbent materials.

## 2. Results and Discussion

### 2.1. Solid-Phase Characterization of Adsorbent Materials

**Figure 1:**
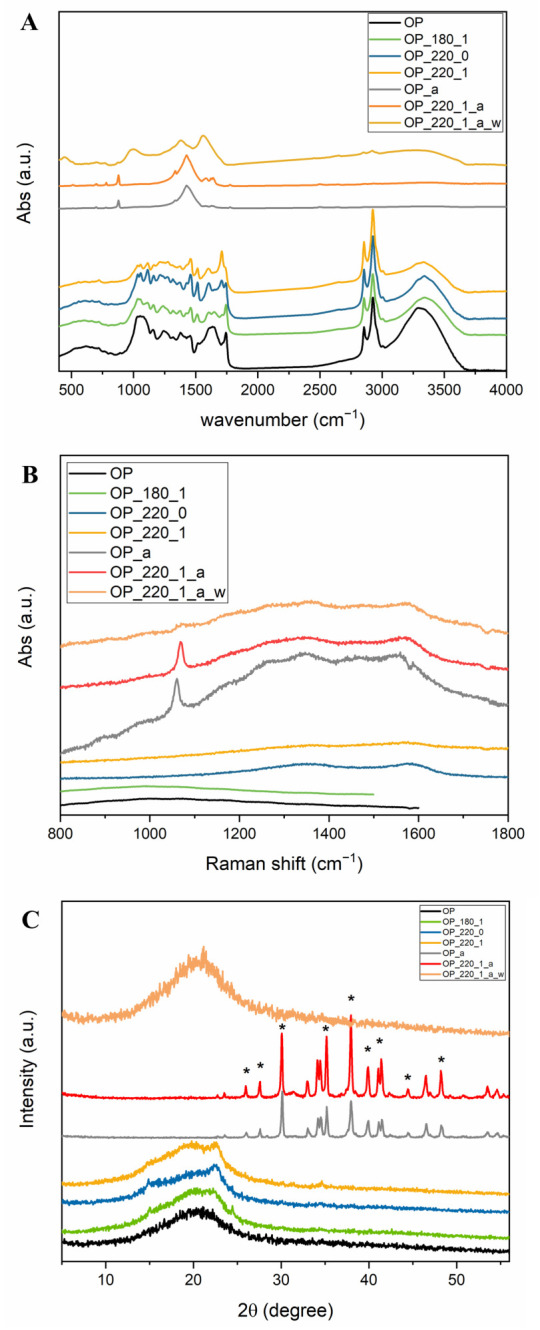
FTIR (**A**), Raman (**B**), and XRD (**C**) spectra of OP materials (raw, unactivated and activated hydrochars) used for MB adsorption. In XRD diffractograms. * = sodium carbonate [00-018-1208].

The ATR-FTIR, Raman, and XRD characterization of the OP materials utilized in this study as adsorbents for MB removal is shown in Figure 1. The ATR-FTIR spectra of raw OP and activated OP adsorbents are shown in Figure 1A. After the HTC treatments, some differences in the chemical structure of the raw material can be observed. The band centered at 3250 cm^−1^, associated with O–H stretching, decreases with increasing HTC temperature, whereas the C–H bands at 2850 and 2920 cm^−1^ remain unchanged. At the same time, the signal corresponding to the stretching vibration of the carbonyl (C=O) group at 1700 cm^−1^ appears after the hydrothermal treatment. The band around 1740 cm^−1^ was assigned to C=O stretching of ester, carboxylic, and residual lipid-related groups. Modifications in the fingerprint region (1000–1600 cm^−1^), associated with C–O, C–O–C and aromatic skeletal vibrations of the lignocellulosic matrix, indicate the progressive degradation of hemicellulose and cellulose together with the formation of a more condensed carbonaceous framework.

The characteristic bands of the carbonate ion stretching vibrations at 870 and 1410 cm^−1^ dominate the spectra of the activated adsorbents, masking the signals of the carbonaceous material and suggesting the precipitation of carbonate during the activation process. Their decrease after washing confirmed carbonate removal from the activated hydrochar surface. The salt was dissolved during the washing step, and the spectrum of the OP_220_1_a_w sample is dominated by broad bands in the 1000–1500 cm^−1^ region, which can be attributed to the carbonaceous backbone, and a broad band around 3300 cm^−1^ assigned to O–H vibrations.

The Raman spectra (Figure 1B) of the raw OP and OP hydrochar prepared at 180 °C are dominated by broad, structureless luminescence bands, confirming the presence of conjugated and hydrogenated functionalities, as also suggested by the ATR-FTIR analysis. The formation of an amorphous carbon-like structure begins at higher temperatures, and in the Raman spectra of hydrochars prepared at 220 °C, the D (defect) and G (graphitic) bands, centered at approximately 1340 and 1580 cm^−1^, respectively, become visible. The activation process appears to strongly affect the carbonaceous structure, as indicated by the shift and quenching of the luminescence observed for the OP and OP_180 samples. However, a signal at 1066 cm^−1^, attributed to the CO_3_^2-^ group, is clearly distinguishable in the Raman spectra of OP_a and OP_220_1_a. The carbonate signal is no longer present after the washing procedure.

The precipitation of carbonate species in the activated hydrochars is confirmed by the XRD analysis (Figure 1C). The diffractograms of raw OP and the hydrochars are dominated by a broad band centered at about 2θ = 20°, confirming the amorphous character of the prepared materials. After activation, new narrow peaks appeared. For the activated samples, all reflections are related to sodium carbonate (JCPDS no. 00-018-1208). The diffractogram of the washed sample confirms the efficient removal of Na_2_CO_3_.

**Figure 2:**
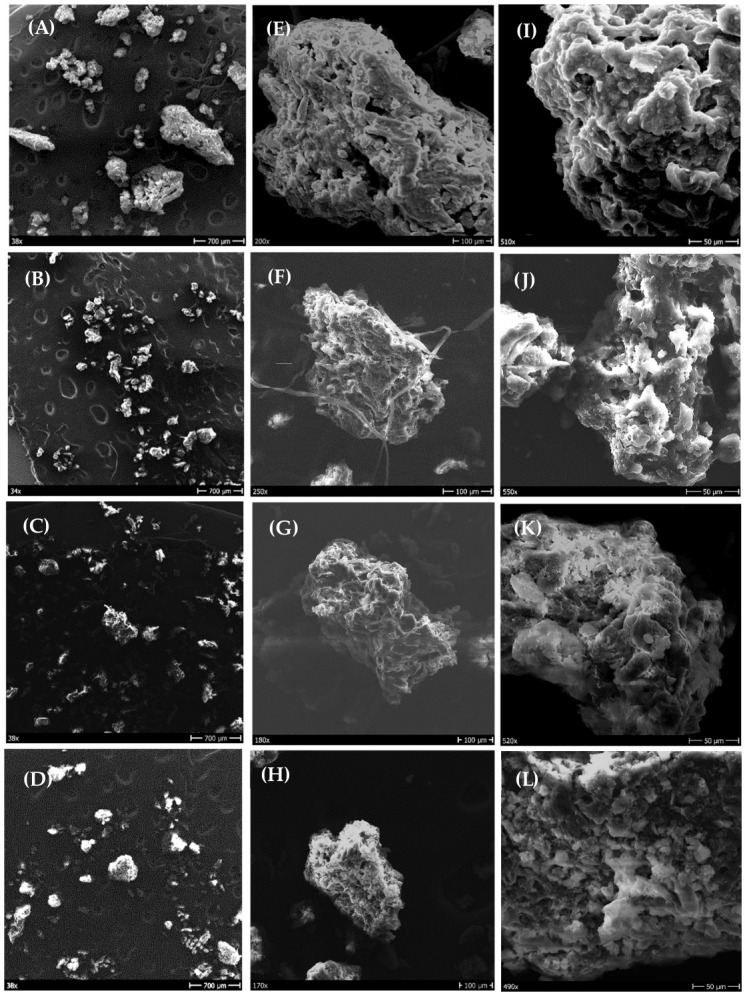
SEM micrographs of raw OP (**A**,**E**,**I**) and hydrochars produced at 180 °C for 1 h, OP_180_1 (**B**,**F**,**J**), 220 °C for 0 h, OP_220_0 (**C**,**G**,**K**), and 220 °C for 1 h, OP_220_1 (**D**,**H**,**L**), acquired at increasing magnifications.

Figure 2 shows SEM micrographs of raw OP (Figure 2A,E,I and of OP hydrochar obtained at 180 °C for 1 h (Figure 2B,F,J), at 220 °C for 0 h (Figure 2C,G,K), and 1 h (Figure 2D,H,L). OP sample contains particles ranging in size from tens to approximately 1000 microns (Figure 2A). The HTC treatment’s main effect is to break up the larger particles present in the OP sample. In fact, all hydrochars (Figure 2B–D) contain particles ranging from tens of microns to 200–300 microns.

The raw OP appears to be a relatively compact material characterized by the presence of macropores ranging from tens of microns to hundreds of microns in size (Figure 2E,I). Heat treatment at 180 °C for 1 h (Figure 2F,J) and at 220 °C for 0 h (Figure 2G,K) did not appear to cause appreciable morphological changes to the particles. Conversely, the hydrochar obtained at 220 °C for 1 h (Figure 2H,L) is characterized by a greater number of pores.

**Figure 3:**
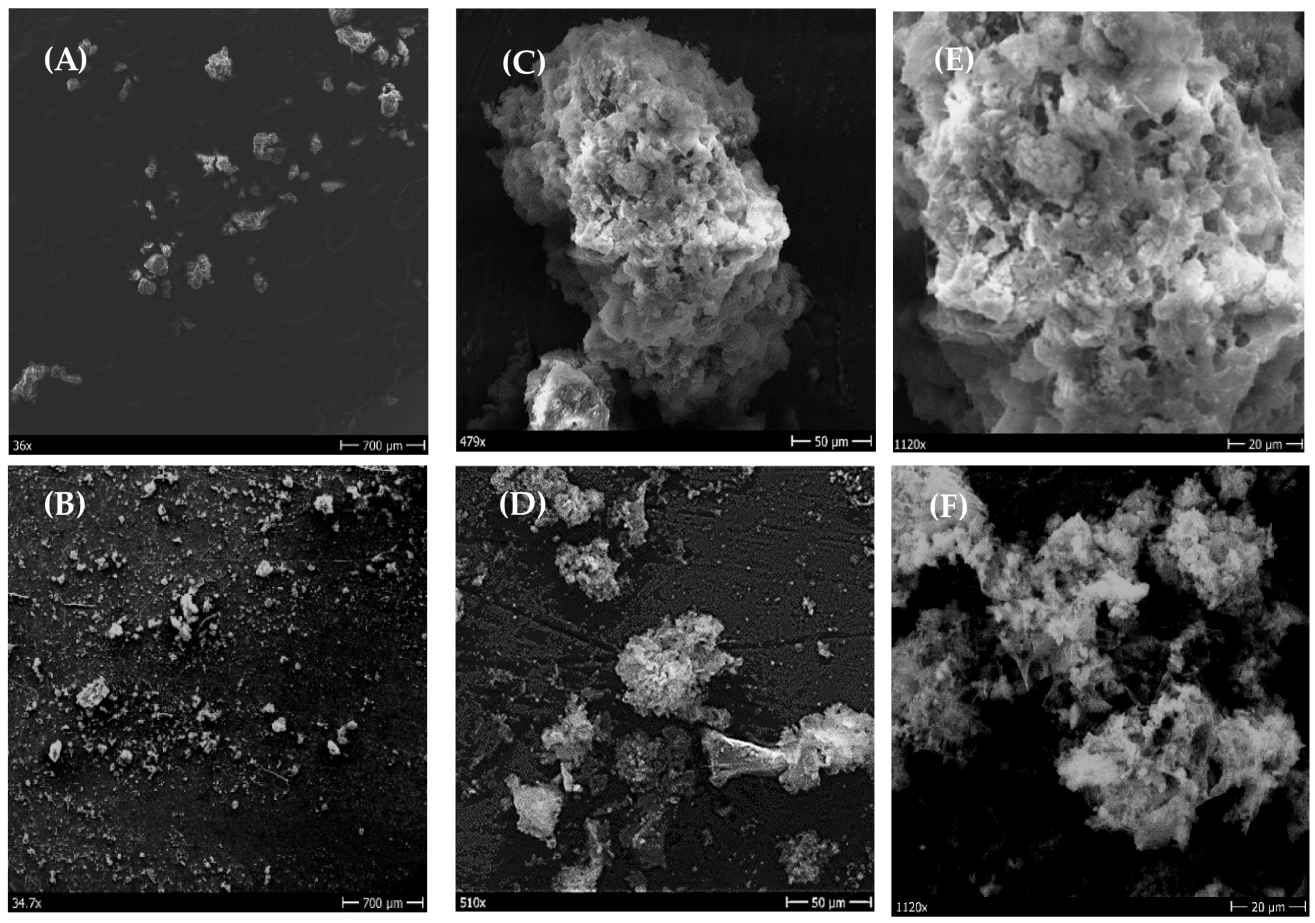
SEM micrographs of SAA-treated raw OP, OP_a (**A**,**C**,**E**), and washed activated hydrochar, OP_220_1_a_w (**B**,**D**,**F**), acquired at increasing magnifications.

The SAA treatment caused further disintegration of the particles (Figure 3A,B) compared to the corresponding non-activated samples (Figure 2A,D). OP_a (Figure 3C,E) was characterized by a smaller number of pores and smaller size than the raw OP (Figure 2E,I), both due to the activation treatment and the precipitation of sodium carbonate. OP_220_1_a_w was characterized by loosely packed secondary particles (Figure 3D) consisting mainly of aggregates of primary plate-like particles (Figure 3F) with the presence of pores of both micrometric and nanometric dimensions. The latter are the result of the collapse of the larger pores present in the non-activated sample following a partial reduction of sodium oxides by carbon with consequent formation of carbon monoxide and sodium carbonate.

Table 1 reports the EDX-derived elemental composition of the investigated materials. Raw OP contained 74.1 wt% C and 21.0 wt% O, corresponding to an O/C ratio of 0.28. Compared with raw OP, the hydrochars showed higher C contents, ranging from 82.5 to 86.5 wt%, and lower O contents, ranging from 11.1 to 15.0 wt%. Consequently, the O/C ratio decreased from 0.28 for raw OP to 0.13–0.18 for the hydrochars. The decrease in the O/C ratio indicates a higher degree of carbonization and a lower abundance of oxygen-containing functional groups.

Following SAA, OP_a and OP_220_1_a contained 36.3 and 36.1 wt% Na, respectively. Compared with the corresponding non-activated materials, SAA decreased the C content and increased both the O content and O/C ratio (Table 1). Washing reduced the O content of OP_220_1_a_w because of the removal of sodium-containing salts. It also has a higher O content and O/C ratio (32.7% and 0.55, respectively) than the non-activated samples and consequently a greater presence of OH functional groups.

Table 1 also reports the BET surface area values for the different materials tested, while Figure S1 shows the pore size distribution curves determined by the BJH method (Figure S1A–D) and by mercury intrusion (Figure S1E–H). Raw OP had a practically negligible volumetric contribution from the micropores and mesopores (Figure S1A). It was characterized by a low surface area, equal to 1.3 m^2^ g^−1^, being a macroporous material with a bimodal porosity distribution (Figure S1E) due to the presence of two peaks characterized by pore radii of 40 and 60 µm, respectively.

Performing HTC at 180 °C for 1 h (OP_180_1) did not determine a volumetric increase in the size distribution of the micro-mesopores (Figure S1B), even if it is included in a larger size range, 0.6 and 10.0 nm (see the enlargement of Figure S1B), compared to raw OP. HTC increased both the macropore volume and the breadth of the pore-size distribution. In fact, OP_180_1 was characterized by a trimodal distribution of porosity (Figure S1F) due to the presence of three peaks with a radius of 25, 50 and 75 µm, respectively. Although the overall pore volume did not increase, the formation of pores of smaller size compared to raw OP contributes to the increase in surface area (2.14 m^2^ g^−1^). The effect of HTC at a higher temperature (220 °C) for 0 h did not cause significant changes in the surface area compared to OP_180_1, confirming the unchanged morphology (Figure 2), while for longer residence times in the HTC reactor (OP_220_1) there was an increase in surface area (3.5 m^2^ g^−1^) due both to an increase in pore volume in the range of 0.6–10 nm (Figure S1C), with an average value of approximately 1.5 nm (see the enlargement of Figure S1C) and to a slight increase in macropores. In fact, the porosimetric curve (Figure S1G) presents a bimodal distribution with average pore radii of 20 and 90 µm, again with a greater volumetric contribution from smaller pores.

The SAA treatment resulted in a significant increase in surface area, equal to 9.51 and 40.04 m^2^ g^−1^ for OP_220_1_a and OP_220_1_a_w, respectively. The increase in surface area, compared to the non-activated materials, is due to the increase in mesopore volume (Figure S1D) at the expense of macropore volume (Figure S1H). These macropores also had an even broader distribution than the non-activated samples, with an average size of 15 and 150 µm, resulting from the formation of carbon oxide and sodium carbonate removed by washing.

### 2.2. Effect of HTC Temperature and Retention Time on Adsorption by OP Hydrochars

**Figure 4:**
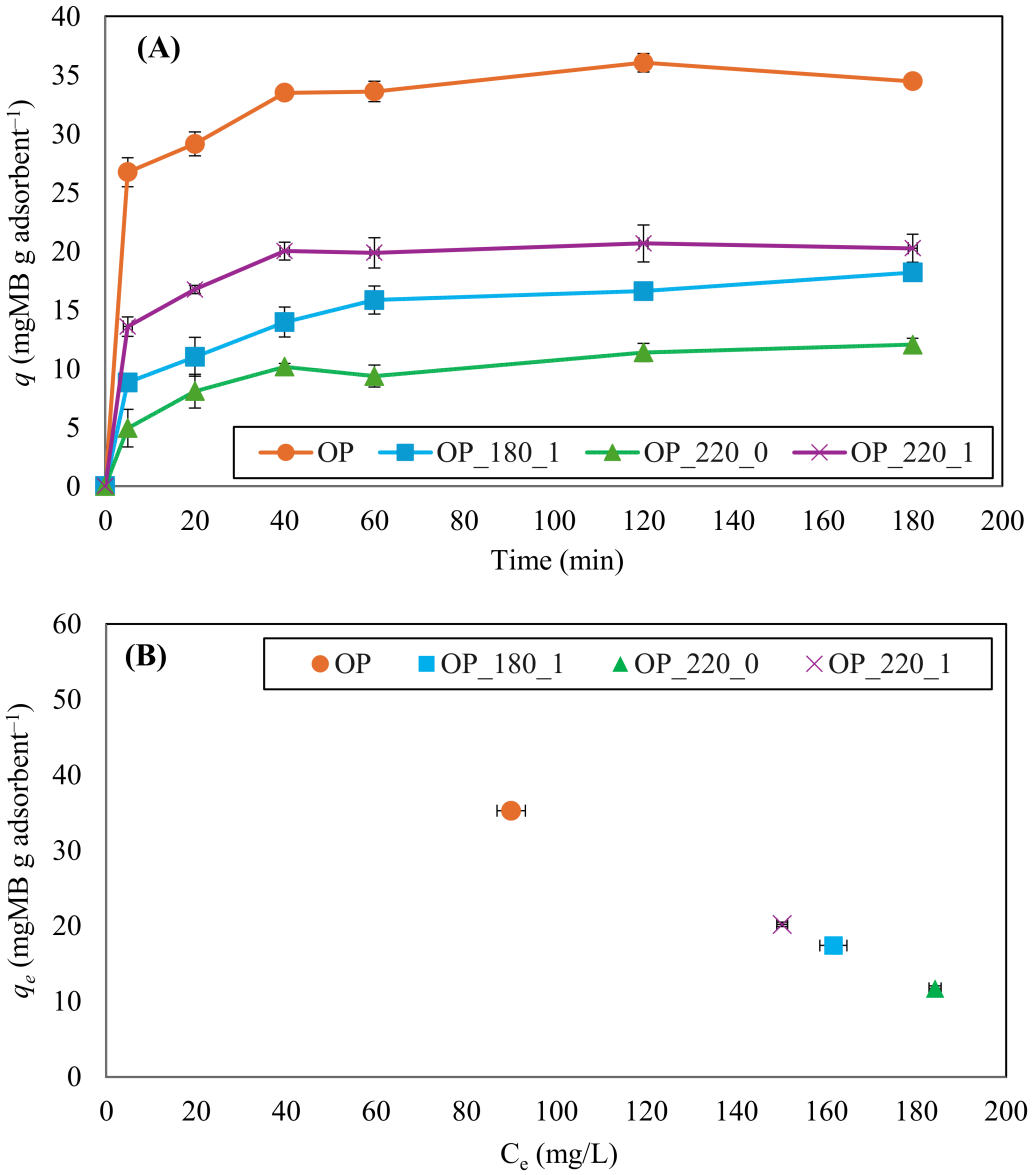
Adsorption capacity profiles (**A**) and equilibrium adsorption data (**B**) for raw OP and hydrochars produced at 180 and 220 °C with residence times of 0 and 1 h. Tests were performed at an initial MB concentration of 250 mg L^−1^ and 20 ± 2 °C.

Figure 4 shows the time profiles of the adsorption capacity of the raw OP sample and its hydrochars obtained at different temperatures (OP_180_1 and OP_220_1) and retention times (OP_220_0 and OP_220_1) (Test 1). The raw OP sample reached the maximum adsorption capacity of 36.1 mg MB g^−1^ adsorbent in 120 min. With OP hydrochar, more than 40% lower adsorption capacities were observed. The best result was obtained by sample OP_220_1, with 20.7 mg MB g^−1^ adsorbent, while lower adsorption capacities were observed for samples OP_180_1 and OP_220_0 (18.2 and 12.1 mg MB g^−1^ adsorbent, respectively).

The greater adsorption capacity of raw OP compared to hydrochars is attributable to the fact that, despite having a smaller surface area (Table 1), it has a higher O/C ratio and therefore a greater number of functional groups that make it more active. The temperatures and retention times used for the HTC treatment also influence the adsorption properties. For the same retention time (1 h), the HTC treatment at higher temperatures improved the adsorption capacity of the produced hydrochar. In fact, OP_220_1, despite having a lower O/C ratio (0.13) and therefore a smaller number of functional groups than OP_180_1 (O/C = 0.18), exhibited a greater adsorption capacity due to the formation of a larger pore volume, which resulted in an increased surface area. For the same HTC temperature (220 °C), the retention time also improved the hydrochar’s adsorption capacity. Comparing the adsorption curves obtained with OP_220_0 and OP_220_1, the latter achieved a higher adsorption capacity. In fact, the absence of a residence period at 220 °C did not induce substantial morphological changes in the produced hydrochar, resulting in a lower adsorption capacity even though the O/C ratio is higher compared to OP_220_1. The greater adsorption capacity of OP_180_1 compared to OP_220_0, having the same surface area, is attributable to the greater quantity of functional groups of this material, which is indeed characterized by a higher O/C ratio (Table 1).

### 2.3. Effect of Soft Alkaline Activation and Washing on Adsorption by OP Adsorbents

**Figure 5:**
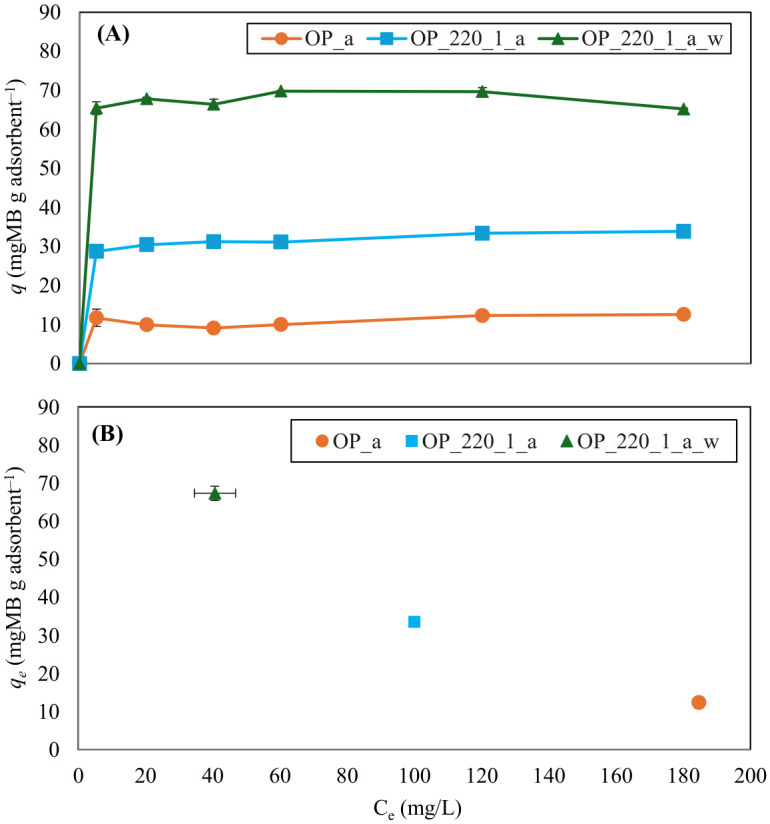
Adsorption capacity profiles (**A**) and equilibrium adsorption data (**B**) for SAA-treated raw OP (OP_a), activated hydrochar (OP_220_1_a), and washed activated hydrochar (OP_220_1_a_w). Tests were performed at an initial MB concentration of 250 mg L^−1^ and 20 ± 2 °C.

The two materials with the best adsorption capacity performance (OP and OP_220_1) were subjected to SAA to evaluate the impact of this activation strategy on adsorption. Following SAA, OP_a and OP_220_1_a were produced. These materials were compared in terms of adsorption capacity at an initial MB concentration of 250 mg L^−1^. Test 2 revealed that, while the adsorption capacity of OP hydrochar was significantly improved by SAA (+63%), the performance of raw OP was compromised, as the adsorption capacity decreased by 65% (Figure 5).

This result contrasts with the observations of Cuccarese et al. [12], who reported a much higher adsorption capacity for SAA-treated spent coffee grounds than those reported for coffee residues in previous studies. OP_220_1_a exhibited a higher adsorption capacity than OP_a because HTC promoted pore development, thereby increasing the specific surface area of the final activated material. This is further enhanced by the activation treatment, in which sodium hydroxide reacts with a material with a higher carbon content (lower O/C ratio), forming more carbonates (as evidenced by a comparison of X-ray diffraction peaks) and carbon monoxide than OP_a. In fact, sample OP_220_1_a has a much higher specific surface area (9.51 m^2^ g^−1^) than OP_a (0.3 m^2^ g^−1^), which explains its higher adsorption performance.

It should be noted that the use of SAA materials raised the pH of the solution by 3–4 units, from 6.5 to 7.0 to 11.0–11.3. Furthermore, SEM and ATR-FTIR observations revealed that the SAA materials were characterized by significant precipitation of sodium carbonates and bicarbonates on their surfaces (Figure 1, Figure 2 and Figure 3), which limited the access of MB to the active sites of the adsorbent. To assess whether the carbonate-rich surface produced during SAA limited MB adsorption, the activated hydrochar was subjected to a post-washing step as described in Section 2.1 and compared with the unwashed material. As can be seen in Figure 5, the *q_e_* of the washed OP hydrochar was approximately 2 times higher than that of OP_220_1_a, revealing that washing improved the adsorption capacity of the activated OP material. This improvement is mainly attributed to the dissolution and removal of carbonate precipitates from the material surface, which reduced pore clogging and restored the accessibility of adsorption sites, as supported by SEM observations and the disappearance of carbonate-related signals after washing (Figure 1, Figure 2 and Figure 3). Although washing also modifies the initial pH conditions, previous investigations on activated hydrochar from spent coffee grounds showed that increasing the pH from 7 to 11 did not exert a significant impact on MB adsorption capacity [19]. Therefore, the higher performance of OP_220_1_a_w is mainly ascribed to improved surface and pore accessibility rather than to pH variation alone.

A similar beneficial effect of SAA was previously observed for spent coffee ground-derived adsorbents [12], including hydrochars [19], confirming that mild alkaline activation can improve the adsorption performance of wet agro-industrial residues.

The relationship between BET surface area and MB adsorption capacity was also evaluated using the *q_e_* values obtained at 20 °C and an initial MB concentration of 250 mg L^−1^. When all OP-derived materials were considered, an apparent positive linear correlation was observed between BET surface area and *q_e_* (Pearson R^2^ = 0.85). However, this relationship was mainly driven by OP_220_1_a_w, which showed both the highest BET surface area (40.04 m^2^ g^−1^) and the highest *q_e_* (69.9 mg g^−1^). When this material was excluded, the correlation became weak (Pearson R^2^ = 0.19), indicating that BET surface area alone cannot explain the adsorption behavior of the investigated materials. This interpretation is also supported by the weak monotonic correlation obtained considering all materials (Spearman’s ρ = 0.37). For instance, untreated OP exhibited a low BET surface area of 1.30 m^2^ g^−1^ but reached a *q_e_* of 35.5 mg g^−1^, whereas OP_220_1 had a higher BET surface area of 3.50 m^2^ g^−1^ but a lower *q_e_* of 20.7 mg g^−1^. Conversely, post-washing increased the BET surface area of the activated hydrochar from 9.51 to 40.04 m^2^ g^−1^ and increased *q_e_* from 34.2 to 69.9 mg g^−1^. BET surface area contributed to MB adsorption only when pore accessibility and surface chemistry were favorable, confirming that adsorption was governed by the combined effect of surface area, accessible porosity, oxygen-containing functional groups, aromatic domains, and carbonate removal rather than by surface area alone. Therefore, in the present OP-derived hydrochar, carbonate precipitation played a particularly relevant role, making post-washing a critical step to restore pore accessibility. The higher adsorption capacity of the washed material compared with all other materials can be attributed to the combination of its high surface area and greater abundance of accessible functional groups (Table 1).

Nevertheless, evaluating the use of unwashed hydrochar is of operational interest, as the washing process as well as the potential recovery of the powdered material in large-scale plants can be time-consuming and result in additional costs and process units, nullifying the economic advantages of using a residual material for environmental purposes. For instance, powdered activated carbon is typically not recovered or regenerated after use, but rather disposed of with the sludge in water treatment processes due to the difficulty in handling fine powders and the prohibitive costs of reactivation. Therefore, one-time use of unwashed activated hydrochar may be seen as a good compromise in terms of economic sustainability and effectiveness.

### 2.4. Kinetic and Isothermal Behavior of OP Adsorbents

Table 2 reports the kinetic and isotherm models that provided the best fit for each OP-derived adsorbent, together with the corresponding model parameters.

Kinetic characterization was performed for all tested OP-derived adsorbents at 20 °C. The pseudo-second order model provided the best fit (R^2^ ≥ 0.995) for both the untreated OP and the OP hydrochars produced at different temperatures and retention times. Fits to the other models, together with the corresponding statistical parameters (R^2^, SSE, RMSE, and χ^2^) are reported in Table S1. Fitting to the pseudo-second-order model suggests that MB adsorption was mainly controlled by the availability of surface adsorption sites and specific adsorbent-adsorbate interactions. This supports the involvement of surface interactions such as hydrogen bonding, electrostatic attraction, and π–π interactions typical of chemisorption [20]. The predominance of chemisorption over physisorption could be expected for the activated hydrochar, as alkaline washing functionalizes the adsorbent surface with hydroxyl (-OH) groups, which can attract the -N(CH_3_)_2_ groups of the MB molecule through hydrogen bonding (O-H⋯N). However, chemisorption was the main kinetic mechanism also describing MB adsorption onto untreated OP. The higher values of *k*_2_ and *q_e_* observed for untreated OP and OP_220_1_a indicate that MB adsorption onto these two materials was faster and more extensive compared to the other materials.

**Figure 6:**
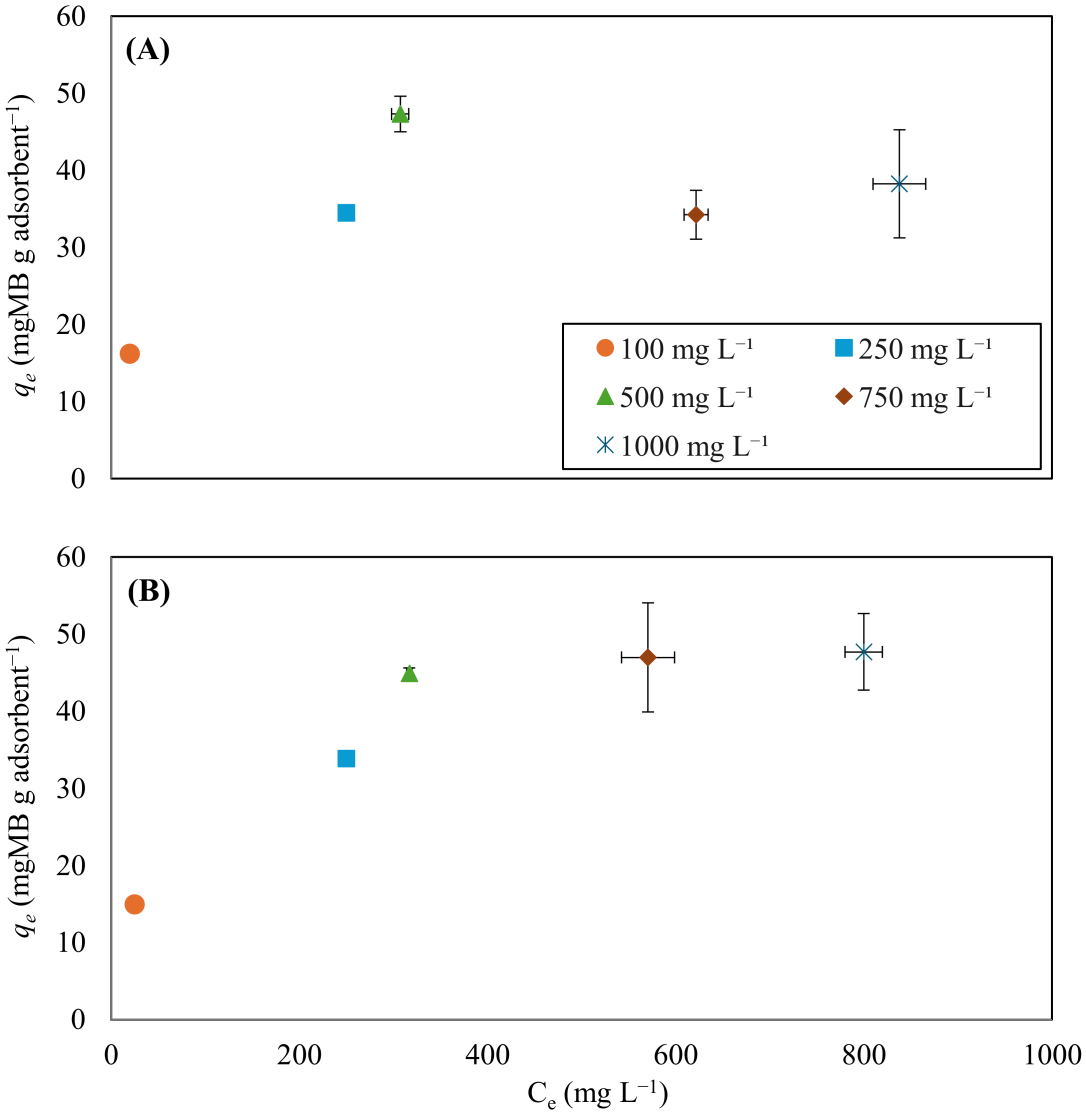
Equilibrium conditions for (**A**) untreated OP and (**B**) activated OP hydrochar produced at 220 °C and 1 h (OP_220_1_a) at 20 °C obtained at different initial MB concentrations (100–1000 mg L^−1^).

The adsorption isotherms were investigated for the best-performing adsorbent materials in terms of *q_e_*, i.e., untreated OP, OP_220_1_a, and OP_220_1_a_w. At 20 °C, OP and OP_220_1_a were well described by the Langmuir isotherm model (R^2^ ≥ 0.98), which describes an adsorption process based on monolayer adsorption with no lateral interaction or transmigration of the adsorbate in the plane of the surfaces and assumes uniform adsorption energies onto the adsorbent surface [21]. Interestingly, the washing process seems to have impacted on the adsorption mechanism, as the best-fitting model shifted from Langmuir to Freundlich for the activated hydrochar at all tested temperatures. The Freundlich model typically indicates multilayer adsorption and can describe adsorption processes on heterogeneous surfaces with potential interactions among the adsorbates and different binding strengths. The binding of multiple MB molecules on each active site can explain the higher adsorption capacity of the washed OP hydrochar. The transition from the Langmuir to the Freundlich isotherm model was also observed at 5 and 40 °C (Table 2) along with the higher *q_e_* of OP_220_1_a_w compared to OP_220_1_a (Figure 6).

### 2.5. Impact of Temperature and Initial Concentration on Adsorption with OP Materials

For OP and OP_220_1_a, the Langmuir isotherm provided the best fit also for adsorption at 5 °C (R^2^ ≥ 0.96) and 40 °C (R^2^ > 0.99), indicating that temperature did not affect the adsorption mechanism (Table 2). For OP_220_1_a, an equally good fit (R^2^ > 0.994) was obtained with the Temkin model. The Temkin isotherm assumes that the adsorption process is characterized by a uniform, infinite energy distribution of the adsorption sites on the adsorbent surface. It also considers the influence of indirect interactions between the adsorbate and the adsorbent on the heat of adsorption of the adsorbed molecules in the layer, which decreases linearly rather than logarithmically (as in the case of Freundlich’s model) with increasing solid surface coverage [22]. Similarly, OP_220_1_a_w was best described by the Freundlich isotherm (R^2^ ≥ 0.95) at different temperatures, confirming that temperature had no effect on the adsorption mechanism in the tested range.

**Figure 7:**
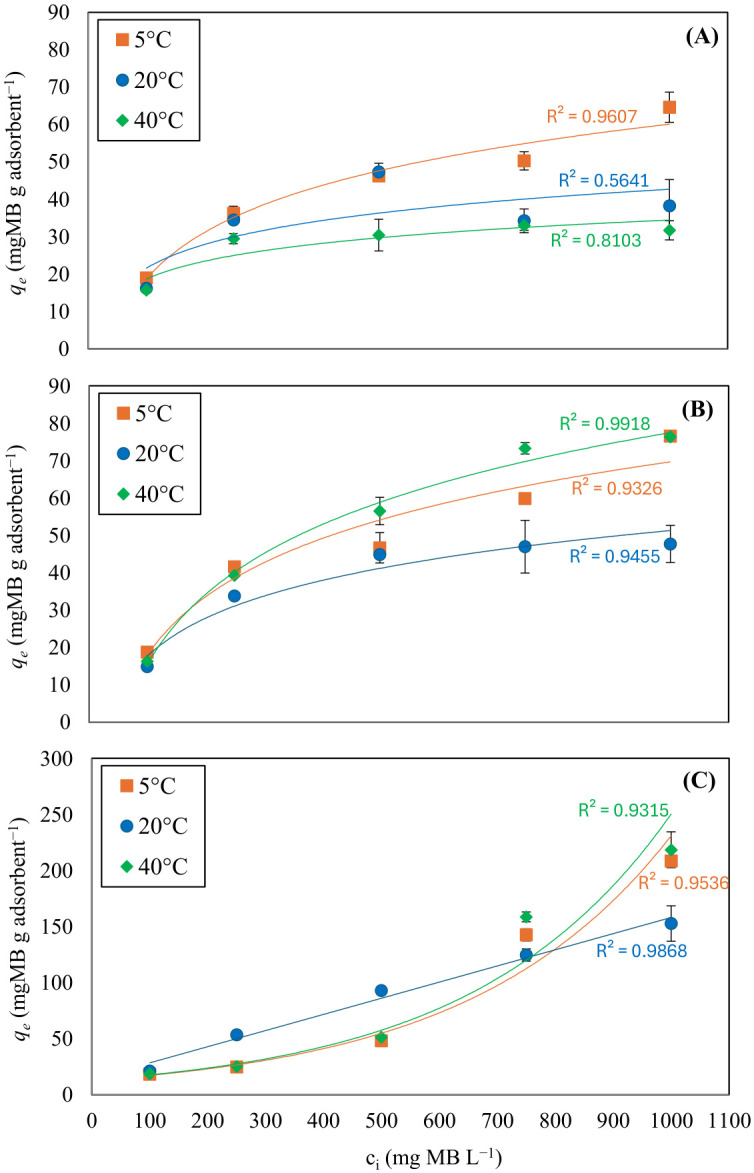
Profiles of adsorption capacity (*q_e_*) vs. initial MB concentration (*c_i_*) at different operational temperatures with (**A**) untreated OP and activated OP hydrochar produced at 220 °C and 1 h before (**B**) and after (**C**) washing.

Figure 7 shows the profiles of *q_e_* at increasing initial MB concentration obtained with the adsorption tests at different temperatures. The adsorption capacity of OP and OP_220_1_a did not change significantly up to 250 mg L^−1^ of MB at the different temperatures tested, while the differences among *q_e_* values were more evident at higher initial MB concentrations. For OP, the *q_e_* profiles at 20 and 40 °C were quite similar except at 500 mg L^−1^ (*p* > 0.05). The highest *q_e_* values were observed at 5 °C and increased with increasing initial MB concentrations. The *q_e_* values at 750 and 1000 mg L^−1^ observed at 5 °C were 1.7–2 times higher than those observed at 20 and 40 °C. For OP_220_1_a, the trends of the adsorption profiles at 5 and 20 °C were similar to those observed for OP, although with slightly higher values.

Much higher adsorption capacities (up to 76.4 mg MB g^−1^ adsorbent) were observed at 40 °C for initial MB concentrations of 500 and 750 mg L^−1^, being also higher than at lower temperatures. Therefore, increasing the temperature had a positive impact on the adsorption performance of the activated hydrochar at high initial MB concentration. The increase in *q_e_* with initial MB concentration with OP and OP_220_1_a was well described by logarithmic equations (R^2^ = 0.81–0.99), except for OP at 20 °C (Table S2). Conversely, the adsorption profiles for OP_220_1_a_w at different temperature fitted well (R^2^ > 0.93) linear (at 20 °C) and exponential functions (at 5 and 40 °C). Such difference in the adsorption profiles at increasing temperature further underlines the superior adsorption capacity of the washed OP hydrochar. Case-specific economic and technical evaluations should therefore be carried out to assess whether ex situ washing and recovery of the activated OP hydrochar powder are advantageous compared to direct use of the unwashed activated material.

### 2.6. Thermodynamics of OP Hydrochar

The thermodynamic behavior of the best-performing OP hydrochar, OP_220_1_a_w, was evaluated at three different temperatures, i.e., 5, 20, and 40 °C, and initial MB concentration of 100 mg L^−1^. The Gibbs free energy (ΔG°) change was calculated according to Equation (3) and remained relatively constant at −3.64(±0.66) kJ/mol in the investigated temperature range, indicating that MB adsorption on the activated and washed OP hydrochar is spontaneous. The values of ΔH° (−14.7 kJ mol^−1^) and ΔS° (−37.6 J mol^−1^ K^−1^) were estimated based on a linear relationship with ΔG° according to Equation (4) (R^2^ = 0.7). The negative ΔH° value indicates an exothermic adsorption process, in which heat is released as MB molecules bind to the hydrochar surface. While the pseudo-second order model indicates that adsorption is controlled by specific surface interactions, the moderate enthalpy change suggests that MB adsorption onto OP-derived hydrochar occurs mainly through weak chemisorptive interactions, including hydrogen bonding and π–π interactions, rather than strong chemical bonding. The negative ΔS° value indicates decreased randomness at the solid–liquid interface because of the restricted mobility of the adsorbed MB molecules.

### 2.7. Preliminary Techno-Economic Considerations Based on Material Efficiency

Beyond adsorption capacity, the material demand required to achieve a given removal target is a key practical driver for the feasibility of adsorption-based treatment. Using the experimental *q_e_* values, the required adsorbent mass to remove 1 kg of MB was calculated as an efficiency indicator (kg adsorbent kg^−1^ MB removed) (Table 3). The material demand indicator was calculated using the *q_e_* obtained from pseudo-second-order kinetic fitting at 20 °C and an initial MB concentration of 250 mg L^−1^ listed in Table 2, ensuring full consistency among the compared materials. The washed activated hydrochar (OP_220_1_a_w) shows the lowest material demand (14.3 kg kg^−1^), corresponding to a 49% reduction compared to untreated OP (28.2 kg kg^−1^) and a 70–82% reduction compared to non-activated hydrochars (48–79 kg kg^−1^). These results further highlight that material washing can significantly improve material efficiency by removing carbonate deposits and increasing the number of accessible adsorption sites. Moreover, they provide a robust and experimentally grounded basis for comparing materials and for highlighting the trade-off between additional processing steps (activation/washing) and reduced adsorbent demand. From an application standpoint, OP-derived adsorbents may be particularly attractive in single-use scenarios where low-cost feedstock and simplified operation can offset lower adsorption capacity compared to commercial activated carbon.

Although the present hydrochars exhibited lower surface areas than conventionally pyrolyzed or intensively activated OP-derived carbons [11], HTC enables the direct processing of wet biomass without an energy-intensive drying step [13]. Therefore, the selection of the conversion route involves a trade-off between maximizing surface area and limiting feedstock pretreatment requirements. However, although HTC avoids energy-intensive feedstock drying, the use of pressurized reactors may increase capital and operating costs. Future studies should therefore compare HTC with lower-pressure thermochemical routes, including wet torrefaction and vapothermal carbonization.

**Figure 8:**
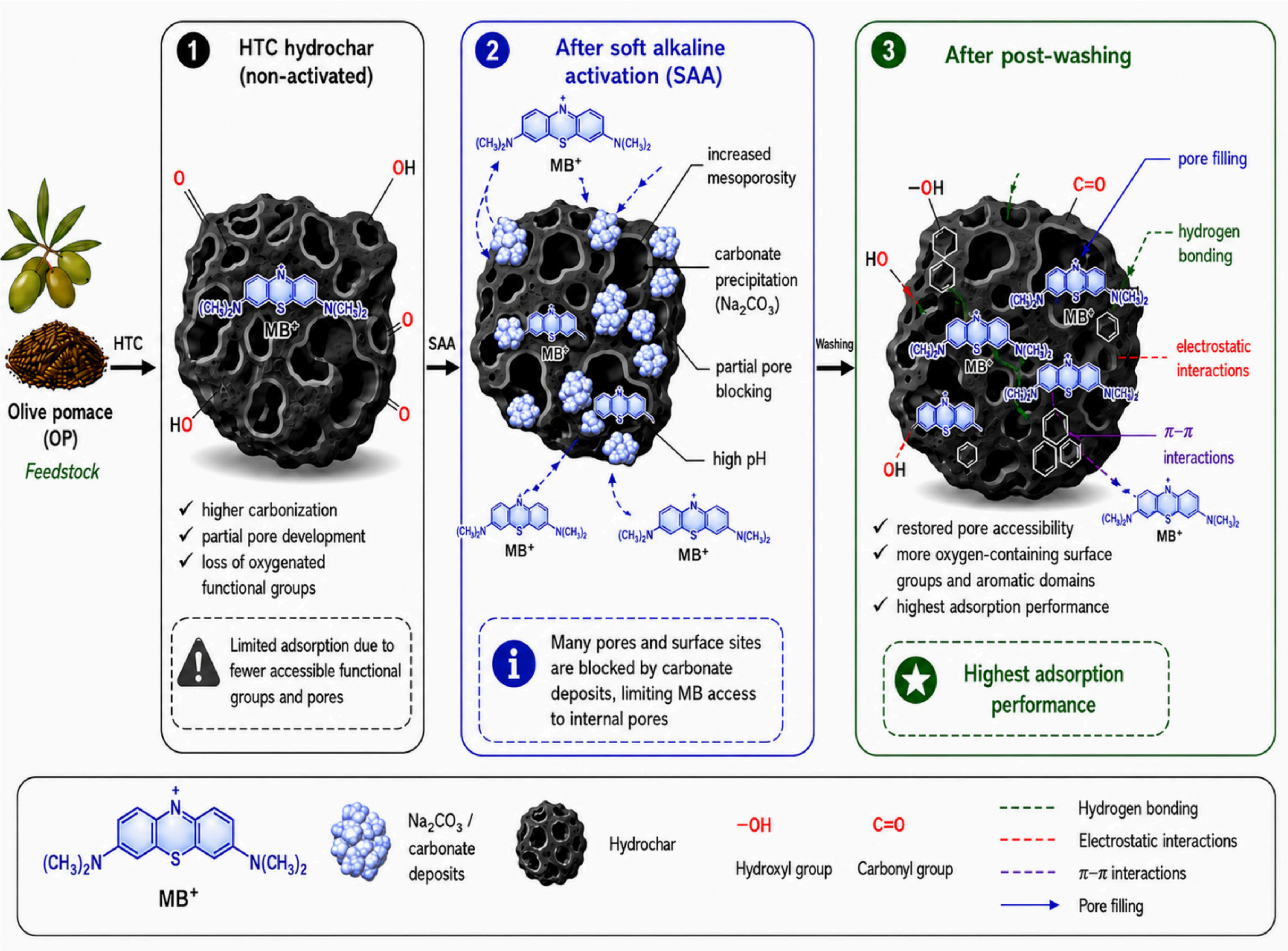
Proposed mechanism of MB adsorption onto OP-derived hydrochars, highlighting HTC-induced carbonization and pore development, carbonate precipitation during SAA, restoration of pore accessibility after washing, and the contribution of pore filling, hydrogen bonding, π–π interactions, and possible electrostatic interactions.

Overall, the combined evidence from spectroscopic, morphological, textural, kinetic and isotherm analyses suggests that MB adsorption onto OP-derived hydrochars was governed by the interplay between surface chemistry and pore accessibility (Figure 8). HTC promoted carbonization and partial pore development, but the concomitant decrease in oxygenated functional groups limited the adsorption capacity of non-activated hydrochars. SAA increased mesoporosity and surface area but also induced sodium carbonate precipitation, which partially blocked pores and adsorption sites. Post-washing removed these carbonate deposits, restored pore accessibility, and exposed oxygen-containing functional groups and aromatic domains. Therefore, MB adsorption onto OP_220_1_a_w can be attributed to the combined contribution of pore filling, hydrogen bonding, possible electrostatic interactions, and π–π interactions rather than to surface area alone. Future pH_pzc_ measurements will be useful to clarify the contribution of surface charge to MB adsorption.

## 3. Materials and Methods

### 3.1. Materials Preparation

OP was collected from an olive mill in northern Sardinia. Due to its inherent moisture content, the OP was used directly without additional water. The material was stored in airtight containers at room temperature until processing. HTC cycles were conducted using the BR-1000 high-pressure reactor (Berghof, Eningen unter Achalm, Germany). HTC cycles consisted of a heating phase, carbonization at the selected temperature (180–220 °C) and residence time (0–1 h), and a cooling phase. The residence time was measured after the reactor reached the target temperature; therefore, 0 h indicates that cooling started immediately after reaching the selected temperature. The OP was processed at 180 °C for 1 h (OP_180_1), 220 °C for 0 h (OP_220_0), and 220 °C for 1 h (OP_220_1). The HTC reactor was loaded with OP, ensuring the correct solid content without additional water. The system was sealed, heated to the target temperature, and maintained for the specified duration. After the reaction, the reactor was allowed to cool naturally before opening. After HTC, the hydrochar and process water were separated using a filter press. The hydrochar was dried at 105 °C overnight and stored in sealed plastic bags. Samples were labeled according to feedstock and processing conditions.

Activated adsorbents (OP_a and OP_220_1_a) were prepared by mixing 5 g of the selected material with 200 mL of NaOH 1 M for 6 h. Subsequently, the impregnated materials were transferred into a ceramic crucible and placed in the oven (TCN 200 PLUS, Argolab, Carpi, Italy) overnight at a temperature of 300 °C. The carbonized materials were then ground using a mortar and pestle and stored.

To evaluate the effect of washing on the adsorption capacity of the activated hydrochar, 7 g of OP_220_1_a was transferred to a 50 mL Falcon tube and manually mixed with 40 mL of distilled water for 2 min. Afterwards, the solution was centrifuged at 4000 rpm for 10 min and the supernatant discarded. The mixing and centrifugation procedure was repeated five times. Afterwards, the pelletized material was placed in a ceramic crucible and dried in the oven at 105 °C overnight. The washed hydrochar material was labelled as OP_220_1_a_w and tested.

### 3.2. Adsorption Tests

The adsorption performance of raw OP and OP-derived materials produced under different HTC and SAA conditions was evaluated in batch tests for MB removal. Comparative, kinetic, isotherm, and thermodynamic adsorption tests were performed on the selected materials. The adsorption tests were performed in triplicate in plastic vials placed on a rotary shaker (Argolab, Carpi, Italy) at 200 rpm. Adsorbent material (200 mg) was added to 50 mL MB solution at different initial MB concentrations. MB (Reag. Ph. Eur., Carlo Erba, Milan, Italy) solutions were prepared in distilled water. The pH of the MB solutions ranged between 6.5 and 7.0. The adsorption capacity *q* was evaluated according to Equation (1):

(1) $$q=\frac{mass\;of\;MB\;adsorbed\;(mg)}{mass\;of\;adsorbent\;(g)}=\left(c_{i}-c_{f}\right)\cdot \frac{V}{m}$$

Table 4 lists the batch tests performed to investigate the adsorption performance of the OP materials.

#### 3.2.1. Comparative Tests

Comparative tests were performed to screen the performance of various OP materials as adsorbents for the removal of MB from water. The first test (Test 1) compared the adsorption capacity of raw OP and OP hydrochars produced at two different temperatures (180 and 220 °C) and retention times (0 and 1 h). The two best-performing materials in terms of adsorption capacity were subjected to SAA and compared in terms of adsorption capacity (Test 2). To evaluate the effect of washing and pH on adsorption by the activated hydrochar, a test comparing the activated OP hydrochar produced at 220 °C before and after the washing procedure was carried out (Test 3). The comparative tests were performed at an MB concentration of 250 mg L^−1^ and temperature of 20 (±2) °C (Table 4). Liquid samples were withdrawn from the vials at different times (0, 5, 20, 40, 60, 120, and 180 min) for MB concentration and q evaluation.

#### 3.2.2. Kinetic and Isotherm Tests

Adsorption kinetics were investigated with batch tests (Test 4) carried out at an initial MB concentration of 250 mg L^−1^ and at 20 (±2) °C. The fitting of experimental data to five different kinetic models (pseudo-first order, pseudo-second order, intraparticle diffusion, liquid film diffusion, and Elovich, see Table 5) was evaluated by collecting the samples at 0, 5, 20, 40, 60, 120 and 180 min.

Adsorption isotherms were evaluated in batch tests (Test 5) with initial MB concentrations of 100, 250, 500, 750, and 1000 mg L^−1^ and at a temperature of 20 (±2) °C. The experimental data were fitted to four different isotherm models (Langmuir, Freundlich, Temkin, and Dubinin–Radushkevich, see Table 5) by collecting the liquid samples at 0 and 180 min. Moreover, the Langmuir isotherm dimensionless constant, *R_L_*, was calculated as follows [23]:

(2) $$R_{L}=\frac{1}{1+K_{L}\cdot C_{0}}$$

Based on the *R_L_* value, the adsorption process is considered favorable (0 < *R_L_* < 1), unfavorable (*R_L_* > 1), irreversible (*R_L_* = 0), or linear (*R_L_* = 1).

#### 3.2.3. Thermodynamic Tests

Adsorption thermodynamics were evaluated by performing batch tests at different temperatures, i.e., 5, 20 and 40 °C (Test 6). Thermodynamic analysis included the investigation of the effect of temperature as well as the Gibbs free energy, enthalpy, and entropy of the process. For the tests performed at 5 and 40 °C, the rotary shaker containing the capped adsorption vials was operated inside a temperature-controlled incubator (FTD ISCO, Lincoln, NE, USA) maintained at the selected temperature throughout the experiment. The MB solutions and adsorption vials were equilibrated at the target temperature for 24 h before adsorbent addition.

The ∆G°, ∆H°, and ∆S° of the adsorption process were calculated using the following equations:

∆G° = −RT ln Ka(3)

∆G° = ∆H° − T ∆S°(4)

By combining Equations (3) and (4) the following equation is obtained:

(5) $$\ln K_{a}=-\frac{∆H{}^{\circ}}{RT}+\frac{∆S{}^{\circ}}{R}$$

*K_a_* can be reasonably approximated by the distribution constant *K_c_* = *C_ad_ C_e_*^−1^ [24]. By plotting ln *K_a_* versus 1/*T* the enthalpy and entropy of the adsorption process can be estimated.

### 3.3. Analytical Methods

MB concentration and pH in the liquid samples were measured as described by Di Capua et al. [25]. Solid materials were analyzed by X-ray diffraction (XRD), Raman and Attenuated Total Reflectance Fourier Transform Infrared (ATR-FTIR) spectroscopy, Scanning Electron Microscopy (SEM), energy-dispersive X-ray (EDX) spectroscopy, Brunauer–Emmett–Teller (BET) analysis and Mercury Intrusion Porosimetry (MIP). XRD spectra were obtained at 2q 5°–60°, step size 0.040°, time per step 4 s with a SIEMENS D5000 diffractometer (Siemens AG, Karlsruhe, Germany) operated at 40 kV and 32 mA, using CuKα radiation, in a q-2q configuration. Raman spectra were acquired with a 600 g mm^−1^ holographic grating, obtaining a resolution of 4 cm^−1^ by using a Jobin-Yvon Horiba LabRam spectrometer (HORIBA Jobin Yvon S.A.S., Villeneuve d’Ascq, France) equipped with an Olympus microscope (Olympus Corporation, Tokyo, Japan) with 10×, 50× and 100× objectives. The excitation source was a He-Ne laser (λ = 632.8 nm). ATR-FTIR analysis was performed with a Bruker Alpha II FTIR-4000 instrument (Bruker Optics GmbH & Co. KG, Ettlingen, Germany) operating in the 400–4000 cm^−1^ spectral range and with a resolution of 4 cm^−1^. The particle morphology was investigated by scanning electron microscopy (SEM) (FEI Quanta 600 FEG, Hillsboro, OR, USA) with a field emission electron gun operated at 25 kV. The specific surface area of the powders was measured by the Brunauer–Emmett–Teller (BET) method and the pore size distribution of the powders by the adsorption–desorption isotherm (BJH method) utilizing nitrogen as the adsorbate after drying at 80 °C for 12 h (Gemini 2375, Micromeritics Instrument Inc., Norcross, GA, USA) and by Mercury Intrusion Porosimetry (MIP) (Micromeritics Autopore III, Micromeritics Instrument Inc., Norcross, GA, USA).

### 3.4. Performance-Based Techno-Economic Assessment

A preliminary techno-economic evaluation was carried out using a performance-based indicator derived exclusively from the experimental adsorption results. The assessment aimed at comparing OP-derived materials in terms of material efficiency under identical operating conditions rather than estimating absolute production costs, which would not be representative at laboratory scale due to non-optimized drying, activation and washing procedures.

The functional unit was defined as 1 kg of MB removed from water. For each adsorbent, the required mass of material to remove the functional unit was calculated from the experimentally determined equilibrium adsorption capacity (*q_e_*) as:

(6) $$m_{ads}=\frac{1}{q_{e}}$$

where *m_ads_* is expressed as kg adsorbent per kg MB removed. This metric was used to support a preliminary comparison among untreated OP, hydrochars, and activated hydrochars, and to contextualize their potential applicability with respect to commercial powdered activated carbon values reported in the literature.

### 3.5. Statistical Analysis

The one-way analysis of variance (ANOVA) was used to assess the statistical difference in the adsorption capacity profiles obtained with the different OP adsorbents. ANOVA was performed with Excel 365 (Microsoft, Redmond, WA, USA). The fitting of the isotherm and kinetic models with experimental data was evaluated according to the coefficient of linear determination (Equation (7)) and other error functions including the sum of the squares of the errors (SSE) (Equation (8)), the residual root mean square error (RMSE) (Equation (9)), and the chi-square test (χ^2^) (Equation (10)).

(7) $$R^{2}=\frac{1-\sum_{n=1}^{n}{\left(q_{e,n}-q_{m,n}\right)}^{2}}{\sum_{n=1}^{n}{\left(Q_{e,n}-Q_{m,n}\right)}^{2}}$$

(8) $$SSE=\sum_{n=1}^{n}{\left(q_{e,n}-q_{m,n}\right)}^{2}$$

(9) $$RMSE=\sqrt{\frac{1}{n-1}\sum_{n=1}^{n}{\left(q_{e,n}-q_{m,n}\right)}^{2}}$$

(10) $$\chi^{2}=\sum_{n=1}^{n}\frac{{\left(q_{e,n}-q_{m,n}\right)}^{2}}{q_{e,n}}$$

## 4. Conclusions

This study demonstrated that OP can be effectively valorized into functional hydrochar-based adsorbents for dye removal from water through the combined application of HTC and SAA. HTC significantly modified the morphology and surface chemistry of the produced materials by increasing the degree of carbonization, promoting pore development, and increasing surface area. However, the reduction of oxygenated functional groups limited the adsorption performance of non-activated hydrochars compared with untreated OP.

Among the HTC conditions investigated, treatment at 220 °C for 1 h produced the best-performing non-activated hydrochar, reaching 20.7 mg g^−1^. SAA increased the adsorption capacity to 34.2 mg g^−1^ and increased the BET surface area to 9.51 m^2^ g^−1^. However, SAA also induced sodium carbonate precipitation, partially blocking pores and adsorption sites. Post-washing removed these carbonate deposits and increased the adsorption capacity from 34.2 to 69.9 mg g^−1^, while the BET surface area increased from 9.51 to 40.04 m^2^ g^−1^.

The washed activated hydrochar showed the lowest adsorbent demand among the investigated materials, requiring 14.31 kg of adsorbent per kg of MB removed, corresponding to a 49% reduction compared with untreated OP. Nevertheless, raw OP also showed a relatively high adsorption capacity (35.5 mg g^−1^) without requiring carbonization, activation, or washing, and may therefore represent a simpler alternative when minimal processing is prioritized. Adsorption kinetics were consistently described by the pseudo-second-order model, while the washed material was better described by the Freundlich isotherm, indicating heterogeneous adsorption behavior. Thermodynamic analysis showed that adsorption was spontaneous and exothermic, with ΔG° = −3.64 ± 0.66 kJ mol^−1^ and ΔH° = −14.7 kJ mol^−1^.

Overall, the results demonstrate that adsorption performance was governed by the combined contribution of surface chemistry, accessible porosity, and surface functional groups rather than by surface area alone. The combination of HTC, SAA, and washing therefore represents a promising approach for producing OP-derived adsorbents, although future studies should optimize material recovery and washing requirements and evaluate regeneration, adsorption of dyes with different molecular structures and charges, and performance under real wastewater conditions.

## Figures and Tables

**Table 1:** EDX-derived elemental composition and BET surface area of OP adsorbent materials.

Sample	C (wt%)	O (wt%)	O/C	Na/K/Cu/Cl/Ca (wt%)	BET Surface Area (m^2^/g)
OP	74.1	21.0	0.28	0.0/4.1/0.4/0.4/0.4	1.30 ± 0.01
OP_180_1	82.5	15.0	0.18	0.0/1.1/0.7/0.3/0.4	2.14 ± 0.09
OP_220_0	84.5	12.6	0.15	0.0/1.6/0.5/0.3/0.4	2.18 ± 0.11
OP_220_1	86.5	11.1	0.13	0.0/1.5/0.3/0.3/0.7	3.50 ± 0.10
OP_a	14.2	45.8	3.22	36.3/2.3/0.9/0.2/0.3	0.3 ± 0.05
OP_220_1_a	20.1	40.3	2.0	36.1/2.6/0.4/0.2/0.3	9.51 ± 0.08
OP_220_1_a_w	59.9	32.7	0.55	0.0/2.1/2.4/0.3/2.6	40.04 ± 0.12

Note: 180 and 220 indicate HTC temperature (°C); 0 and 1 indicate residence time (h); “a” indicates soft alkaline activation; and “w” indicates post-washing.

**Table 2:** Best kinetic and isotherm models for adsorption with the tested OP-derived adsorbent materials at 20 °C.

Material	Best Model	R^2^	Model Parameters
**Kinetics (20 °C)**			
OP	Pseudo-second order	0.999	*q_e_* = 35.5 mg g^−1^ *k*_2_ = 0.012 g mg^−1^ min^−1^
OP_180_1	Pseudo-second order	0.996	*q_e_* = 19.0 mg g^−1^ *k*_2_ = 0.004 g mg^−1^ min^−1^
OP_220_0	Pseudo-second order	0.995	*q_e_* = 12.6 mg g^−1^ *k*_2_ = 0.007 g mg^−1^ min^−1^
OP_220_1	Pseudo-second order	0.999	*q_e_* = 20.7 mg g^−1^ *k*_2_ = 0.018 g mg^−1^ min^−1^
OP_220_1_a	Pseudo-second order	0.999	*q_e_* = 34.2 mg g^−1^ *k*_2_ = 0.010 g mg^−1^ min^−1^
OP_220_1_a_w	Pseudo-second order	1.000	*q_e_* = 69.9 mg g^−1^ *k*_2_ = 0.017 g mg^−1^ min^−1^
**Isotherms (20 °C)**			
OP	Langmuir	0.983	*K_L_* = 0.350 L mg^−1^ *q_m_* = 37.5 mg g^−1^ *R_L_* = 0.003
OP_220_1_a	Langmuir	1.000	*K_L_* = 0.019 L mg^−1^ *q_m_* = 51.0 mg g^−1^ *R_L_* = 0.053
OP_220_1_a_w	Freundlich	0.977	*K_F_* = 7.27 mg^1−1/n^ L^1/n^ g^−1^ *n_F_* = 1.97
**Isotherms (40 °C)**			
OP	Langmuir	0.998	*K_L_* = 0.038 L mg^−1^ *q_m_* = 33.2 mg g^−1^ *R_L_* = 0.027
OP_220_1_a	Langmuir	0.995	*K_L_* = 0.007 L mg^−1^ *q_m_* = 91.7 mg g^−1^ *R_L_* = 0.140
	Temkin	0.994	*A* = 0.081 L mg^−1^ *b_T_* = 12,496 J mol^−1^
OP_220_1_a_w	Freundlich	0.945	*K_F_* = 2.41 mg^1−1/n^ L^1/n^ g^−1^ *n_F_* = 1.49
**Isotherms (5 °C)**			
OP	Langmuir	0.960	*K_L_* = 0.009 L mg^−1^ *q_m_* = 64.5 mg g^−1^ *R_L_* = 0.087
OP_220_1_a	Langmuir	0.963	*K_L_* = 0.007 L mg^−1^ *q_m_* = 79.4 mg g^−1^ *R_L_* = 0.110
OP_220_1_a_w	Freundlich	0.953	*K_F_* = 2.15 mg^1−1/n^ L^1/n^ g^−1^ *n_F_* = 1.25

Note: 180 and 220 indicate HTC temperature (°C); 0 and 1 indicate residence time (h); “a” indicates soft alkaline activation; and “w” indicates post-washing.

**Table 3:** Performance-based material demand for MB removal with OP-derived adsorbents.

Material	*q_e_* (mg g^−1^)	Adsorbent Demand (kg Adsorbent kg^−1^ MB Removed)	HTC Solid Yield (g Hydrochar g^−1^ OP)	Raw Material Demand (kg Raw OP kg^−1^ MB Removed)
OP	35.5	28.17	-	28.17
OP_180_1	19.0	52.63	0.73	72.23
OP_220_0	12.6	79.37	0.84	94.49
OP_220_1	20.7	48.31	0.63	76.94
OP_220_1_a	34.2	29.24	0.63	46.57
OP_220_1_a_w	69.9	14.31	0.63	22.79

Notes: (1) For activated and washed materials, raw OP demand was estimated based on HTC solid yield only; additional mass losses during activation and washing were not included. (2) Sample code: 180 and 220 indicate HTC temperature (°C); 0 and 1 indicate residence time (h); “a” indicates soft alkaline activation; and “w” indicates post-washing.

**Table 4:** Adsorption tests performed with OP and OP hydrochars.

Test	Materials	MB Concentration (mg L^−1^)	Temperature (°C)
1—Effect of HTC operation	OP OP_180_1 OP_220_0 OP_220_1	250	20
2—SAA effect	OP_a OP_220_1_a	250	20
3—Effect of washing	OP_220_1_a OP_220_1_a_w	250	20
4—Kinetics	OP OP_180_1 OP_220_0 OP_220_1 OP_220_1_a OP_220_1_a_w	250	20
5—Isotherms and effect of initial MB concentration	OP OP_220_1_a OP_220_1_a_w	100, 250, 500, 750, 1000	20
6—Effect of temperature	OP OP_220_1_a OP_220_1_a_w	100, 250, 500, 750, 1000	5, 20, 40
7—Thermodynamics	OP_220_1_a_w	100	5, 20, 40

Note: 180 and 220 indicate HTC temperature (°C); 0 and 1 indicate residence time (h); “a” indicates soft alkaline activation; and “w” indicates post-washing.

**Table 5:** Kinetic and isotherm models considered in this study to describe adsorption.

Kinetic Models	Model Equation
Pseudo-first order	$log\left(q_{e}-q_{t}\right)=log\;\left(q_{e}\right)-k_{1}t$
Pseudo-second order	$\frac{t}{q_{t}}=\frac{1}{k_{2}q_{e}^{2}}+\frac{t}{q_{e}}$
Elovich	$q_{t}=\frac{1}{\beta }ln\left(\alpha \beta \right)+\frac{1}{\beta }ln(t)$
Liquid film diffusion	$ln\left(1-\frac{q_{t}}{q_{e}}\right)=-k_{fd}t$
Intraparticle diffusion	$q_{t}=k_{dif}t^{1/2}+C$
**Isotherm models**	
Langmuir	$\frac{C_{e}}{q_{e}}$= $\frac{1}{K_{L}q_{m}}$+ $C_{e}/q_{m}$
Freundlich	$\ln q_{e}=\ln K_{f}+\frac{1}{n_{F}}lnC_{e}$
Temkin	$\frac{q_{e}}{q_{m}}=\frac{RT}{b_{T}}\ln AC_{e}$
Dubinin–Radushkevich	$\ln\left(q_{e}\right)=\ln\left(q_{s}\right)-\beta_{DR}{\;\varepsilon }^{2}$ $\varepsilon =RTln(1+\frac{1}{C_{e}})$ $E=\frac{1}{-\sqrt{2\beta_{DR}}}$

## Data Availability

Data will be made available on request.

## Supplementary Materials

The following supporting information can be downloaded at: https://www.mdpi.com/article/10.3390/molecules31152617/s1, Table S1: Statistical parameters obtained from data analysis of kinetic and isotherm studies; Table S2: Fitting of mathematical functions describing the increase in the adsorption capacity with the initial MB concentration. Figure S1: Pore size distribution curves of samples OP (a,e), OP_180_1 (b,f), OP_220_1 (c,g) and OP_220_1_a_w (d,h) obtained by BJH method (a–d) and by mercury intrusion (e–h).

## Nomenclature

The following nomenclature is used in this manuscript:

*q*	adsorption capacity (mg g^−1^)
*V*	initial volume of the solution for the tests (L)
*m*	adsorbent weight (g)
*m_ads_*	adsorbent demand (kg adsorbent kg^−1^ MB removed)
*c_i_*	MB concentration at the beginning of the test (mg L^−1^)
*c_f_*	MB concentration at the end of the test (mg L^−1^)
*q_e_*	equilibrium adsorption capacity (mg g^−1^)
*q_t_*	adsorption capacity at time t (mg g^−1^)
*k*_1_	rate constant of pseudo-first order (min^−1^)
*k*_2_	rate constant of pseudo-second order (g mg^−1^ min^−1^)
*K_c_*	distribution constant (dimensionless)
*α*	initial adsorption rate constant (mg g^−1^ min^−1^)
*β*	activation energy constant for chemisorption (g mg^−1^)
*ε*	Polanyi potential (J mol^−1^)
*k_fd_*	liquid film rate diffusion constant (min^−1^)
*k_dif_*	rate constant of intraparticle diffusion (mg g^−1^ min^−1/2^)
*C_e_*	concentration of adsorbate at equilibrium (mg L^−1^)
*C_ad_*	MB concentration of solute adsorbed at equilibrium (mg L^−1^)
*C*_0_	the highest initial MB concentration (mg L^−1^)
*C*	intraparticle diffusion intercept (mg g^−1^)
*K_L_*	Langmuir equilibrium constant (L mg^−1^)
*q_m_*	maximum adsorption capacity (mg g^−1^)
*K_F_*	Freundlich’s constant (mg g^−1^)
*n_F_*	Freundlich heterogeneity factor (dimensionless)
*A*	Temkin isotherm constant (L mg^−1^)
*b_T_*	Temkin constant related to the heat of adsorption (J mol^−1^)
*E*	free energy (kJ mol^−1^)
*q_s_*	theoretical saturation capacity (mg g^−1^)
*β_DR_*	Dubinin–Radushkevich constant (mol^2^ J^−2^)
Δ*G*°	standard free energy (kJ mol^−1^)
Δ*H*°	standard enthalpy (kJ mol^−1^)
Δ*S*°	standard entropy (J mol^−1^ K^−1^)
*T*	temperature (K)
*K_a_*	thermodynamic equilibrium constant (dimensionless)
*R*	gas constant (8.314 J mol^−1^ K^−1^)
*R_L_*	Langmuir isotherm constant (dimensionless)
*pH_pzc_*	pH Point of Zero Charge
