# Supplementary material for: Valorization of Olive Pomace into Functional Hydrochars for Dye Removal from Water: Effects of Hydrothermal Carbonization and Soft Alkaline Activation
Source: Molecules. 2026 Jul 27;31(15):2617. doi: 10.3390/molecules31152617 (PMC13468592; doi:10.3390/molecules31152617)
Supplement: Supplementary file 1 [file molecules-31-02617-s001.zip › molecules-4442009-supplementary.pdf]

molecules

Supplementary Materials

Valorization of olive pomace into functional hydrochars for dye removal from water: effects of hydrothermal carbonization and soft alkaline activation

Gianluigi Farru[1], Gennaro Pace[2], Antonio Di Virgilio[2], Fabiano Asunis[1], Angela De Bonis[3], Maria Cristina Mascolo[4,5,6], Salvatore Masi[2], Francesco Di Capua[2,*]

[1] Department of Civil and Environmental Engineering and Architecture, University of Cagliari, Via Marengo 2, 09123 Cagliari, Italy; gianluigi.farru@unica.it (G.F.); fabiano.asunis@unica.it (F.A.)
[2] Department of Engineering, University of Basilicata, Via dell'Ateneo Lucano 10, 85100 Potenza, Italy; gennaro.pace@unibas.it (G.P.); salvatore.masi@unibas.it (S.M.)
[3] Department of Basic and Applied Science, University of Basilicata, Via dell'Ateneo Lucano 10, 85100 Potenza, Italy; angela.debonis@unibas.it
[4] Department of Letter and Philosophy, University of Cassino and Southern Lazio, Via S. Angelo—Località Folcara, 03043 Cassino, Italy; mc.mascolo@unicas.it
[5] Materials Laboratory, Department of Civil and Mechanical Engineering, University of Cassino and Southern Lazio, Via Gaetano di Biasio 43, 03043 Cassino, Italy
[6] Institute of Environmental Geology and Geoengineering, National Research Council, Area della Ricerca di Roma 1, Via Salaria Km 29.300, C.P. 10, Monterotondo Stazione, 00015 Rome, Italy
* Correspondence: francesco.dicapua@unibas.it

Table of Contents

Table S1 – Statistical parameters obtained from data analysis of kinetic and isotherm studies. 2

Table S2 – Fitting of mathematical functions describing the increase of the adsorption capacity with the initial MB concentration. 3

Figure S1 – Pore size distribution curves of samples OP (A,E), OP_180_1 (B,F), OP_220_1 (C,G) and OP_220_1_a_w (D,H) obtained by BJH method (A-D) and by mercury intrusion (E-H). 4

molecules

MDPI

Table S1 – Statistical parameters obtained from data analysis of kinetic and isotherm studies.

Material	Model	R^2	SSE	RMSE	χ^2
Kinetic tests					
OP	Pseudo-second order	0.999	15.963	1.787	0.557
OP_180_1	Pseudo-second order	0.996	12.100	1.556	1.302
OP_220_0	Pseudo-second order	0.995	3.106	0.788	0.469
OP_220_1	Pseudo-second order	0.999	2.894	0.761	0.168
OP_220_1_a	Pseudo-second order	0.999	58.967	3.434	2.045
OP_220_1_a_w	Pseudo-second order	1.000	37.657	3.068	0.574
Isotherm tests					
OP	Langmuir	0.983	392.079	9.900	19.566
OP 40°C	Langmuir	0.998	10.033	1.584	0.495
OP 5°C	Langmuir	0.960	148.253	6.088	3.452
OP_220_1_a	Langmuir	1.000	3.691	0.961	0.185
OP_220_1_a 40°C	Langmuir	0.995	27.335	2.614	0.525
OP_220_1_a 40°C	Temkin	0.994	14.867	1.928	0.246
OP_220_1_a 5°C	Langmuir	0.963	127.932	5.655	2.329
OP_220_1_a_w	Freundlich	0.977	335.198	9.154	3.848
OP_220_1_a_w 5°C	Freundlich	0.954	1865.137	21.594	18.155
OP_220_1_a_w 40°C	Freundlich	0.944	2197.541	23.439	23.535

Table S2 – Fitting of mathematical functions describing the increase of the adsorption capacity with the initial MB concentration.

Material	T (°C)	Function type	Equation	R^2
OP	5	Logarithmic	$q_e = 17.986 \ln c_i - 64.176$	0.96
	20	Logarithmic	$q_e = 9.1787 \ln c_i - 20.742$	0.56
	40	Logarithmic	$q_e = 6.8544 \ln c_i - 12.911$	0.81
OP_220_1_a	5	Logarithmic	$q_e = 22.452 \ln c_i - 85.408$	0.93
	20	Logarithmic	$q_e = 14.587 \ln c_i - 49.471$	0.95
	40	Logarithmic	$q_e = 26.929 \ln c_i - 108.5$	0.99
OP_220_1_a_w	5	Exponential	$q_e = 13.026\ e^{0.0029\ c_i}$	0.95
	20	Linear	$q_e = 0.144\ c_i + 14.215$	0.99
	40	Exponential	$q_e = 13.252\ e^{0.0029\ c_i}$	0.93

molecules

MDPI

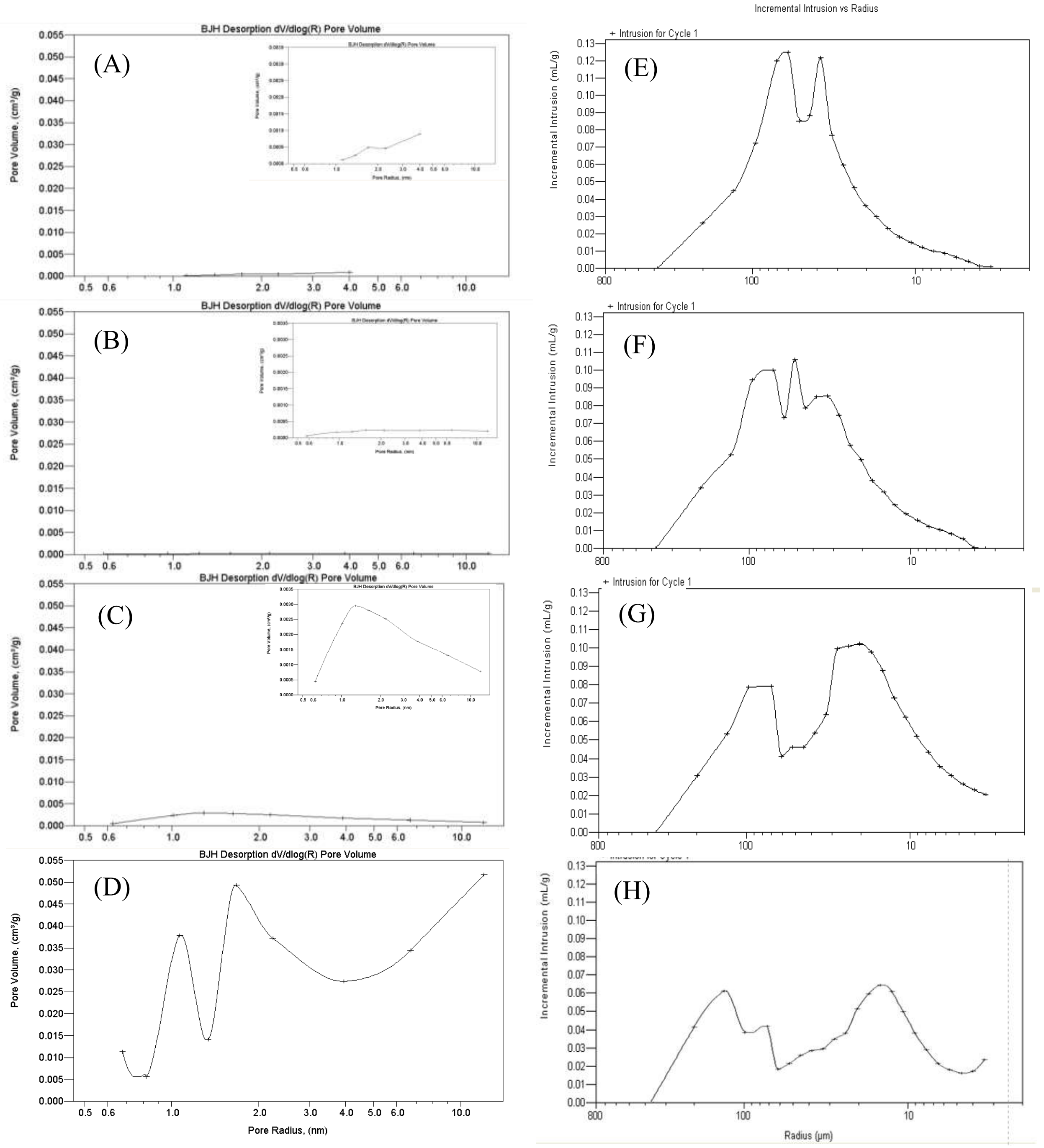

Figure S1 – Pore size distribution curves of samples OP (A,E), OP_180_1 (B,F), OP_220_1 (C,G) and OP_220_1_a_w (D,H) obtained by BJH method (A-D) and by mercury intrusion (E-H).
